# Warty (condylomatous) carcinoma of the back: a case report

**DOI:** 10.1093/jscr/rjab173

**Published:** 2021-05-05

**Authors:** Ilou Park, Sung Hoon Koh, Hee Jeong Lee

**Affiliations:** Department of Plastic and Reconstructive Surgery, Gwangmyeong Sungae General Hospital, Gwangmyeong, Korea; Department of Plastic and Reconstructive Surgery, Gwangmyeong Sungae General Hospital, Gwangmyeong, Korea; Department of Pathology, Gwangmyeong Sungae General Hospital, Gwangmyeong, Korea

## Abstract

Warty carcinoma (WC), known as condylomatous carcinoma, generally derives from genito-urethral area. Its symbolic lesion is the exophytic and verruciform mass associated with human papillomavirus infection. A 90-year-old female presented with growing cauliflower-like mass in her back. A wide excision was performed for two masses. It was finally confirmed as WC throughout histopathological findings—arborescent papillomatosis, hyperkeratosis and acanthosis. The patient was an ordinary housewife and there was no recurrence and any postoperative complication 6 month after the surgery. Accordingly, careful physical examination and history-taking as well as wide-excision securing safety margin are essential, especially for senile patients.

## INTRODUCTION

Warty carcinoma (WC) is a verruciform tumor associated with human papillomavirus (HPV) infection [1, 2]. It is an unusual type of variant squamous cell carcinomas (SCCs) and usually occurs on genito-urethral area such as penis, vulva and uterine cervix and perineum including anus [3]. Most patients complain of a growing mass on the body surface with ulceration, discharge and bleeding. It is reported that the typical microscopic findings are hyperkeratosis, arborescent papillomatosis, acanthosis and prominent koilocytosis with nuclear pleomorphism [4]. WC is known to have less aggressive progression compared with other kinds of SCCs [5]. We present a rare case of 90-year-old female with WC occurring on the back.

**Figure 1:**
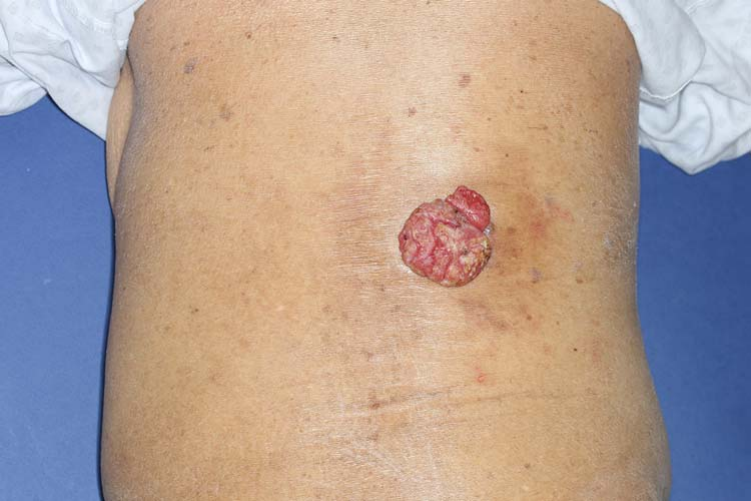
Preoperative photograph. An exophytic and cauliflower-like mass is seen on the back side of approximately T10 level.

**Figure 2:**
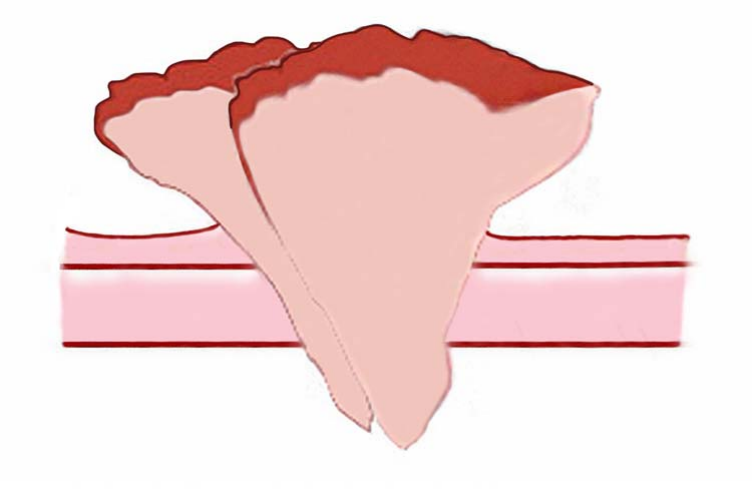
Illustration of the warty carcinoma. Two masses are pedunculated side by side on the back.

**Figure 3:**
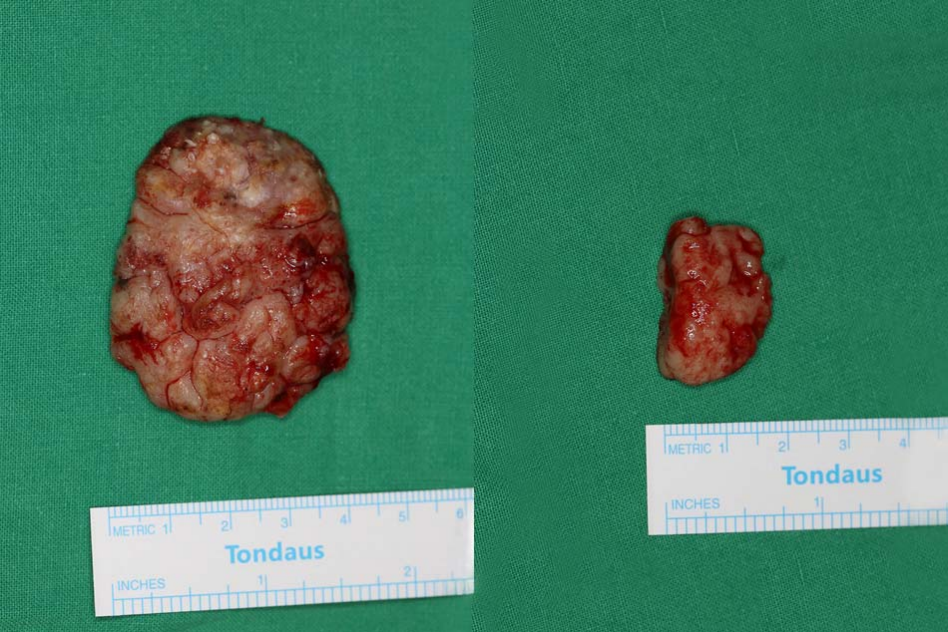
Gross specimen photographs after wide excision. Two masses are completely removed.

**Figure 4:**
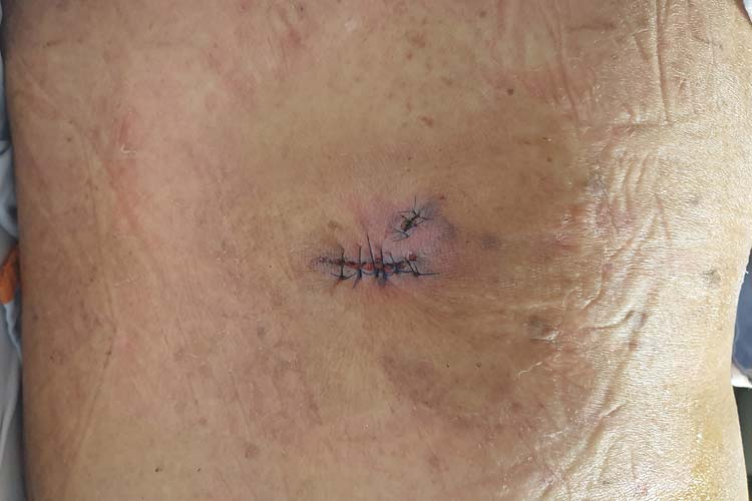
Immediate postoperative photograph. The surgical wound is primarily closed.

**Figure 5:**
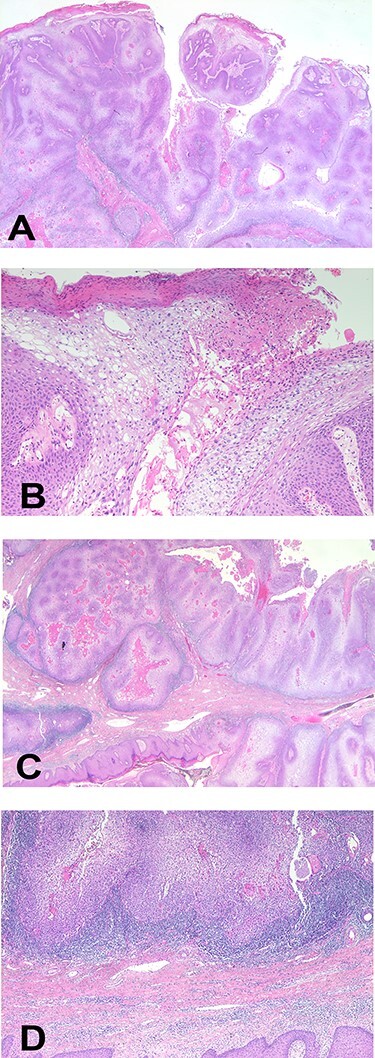
Histopathological images of the mass. (**A**) Arborizing papillomatosis with acanthosis (H&E, ×40), (**B**) parakeratosis of superficial layer, cytoplasmic vacuole and prominent fibrovascular cores (H&E, ×200), (**C**) broadly cancerous stromal invasion (H&E, ×10), (**D**) pleomorphic nuclei with prominent nucleoli and coarse chromatins (H&E, ×10). H&E, hematoxylin and eosin.

**Figure 6:**
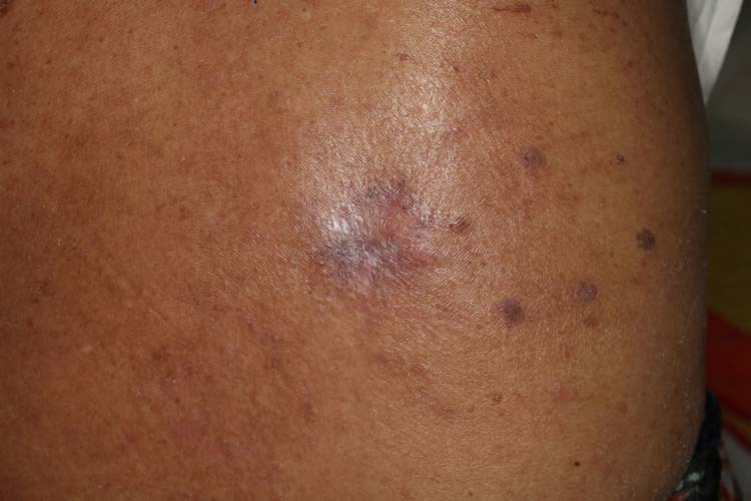
Six-month follow-up photograph. Any complication is not shown on the wound.

## CASE

A 90-year-old woman visited our clinic with cauliflower-like mass on her back. It was insidious in onset and gradually progressive, growing from 3 cm on the first visit to 8 cm after 9 months. Her general condition was fair, except weakness of lower extremities owing to senility. She was an ordinary housewife without any special family history and had total hysterectomy in gynecological history because of uterine leiomyoma. Cytological study revealed positive HPV in previous hysterectomy. All the vitals were stable and there was no palpable lymphadenopathy. Local physical examination revealed a verrucous and pedunculated mass on the back (Fig. 1). The masses were two, in which the larger was 5.0 × 4.0 cm^2^ on the inferomedial side and the smaller was 3.0 × 2.0 cm^2^ on the superolateral side. They invaded into subcutaneous layer (Fig. 2) and were excised widely with 5 mm free margin on the basis of the surgical treatment of other SCCs (Fig. 3) [6]. The incision wound could be primarily closed with Nylon #4-0 (Fig. 4). The gross specimen had ulcerative warty shape, and uninvolved surgical margins were confirmed. The microscopic finding showed arborescent papillomatosis with prominent fibrovascular core, parakeratosis, acanthosis and koilocytosis with nuclear pleomorphism (Fig. 5). Therefore, it was diagnosed as warty (condylomatous) carcinoma. The patient was fine without any complication and there was no recurrence on follow-up after 6 months (Fig. 6).

## DISCUSSION

WC, also called condylomatous carcinoma, is low-grade verruciform tumor, usually identical to vulvar, cervical or anal counterparts [3]. It may be less aggressive than well-differentiated SCCs. A total of 22–100% of them are related to HPV, usually HPV 16 [2]. Its typical lesion is exophytic, white-tan and verruciform mass. Deep invasion is a poor prognostic factor, in common with other SCCs.

The cause of this disease is unknown, but penile WC makes up 7–10% of total penile carcinomas and 34–36% of verruciform group of neoplasms [7]. Its clinical features include (1) slow growing; (2) 17–18% of lymph node metastasis in penile WC; (3) recurrence due to inappropriate excision or multicentric disease not identified at time of surgery; and (4) low mortality rate (0–9%) [4, 8].

Histopathological descriptions are as follow: (1) low-grade verruciform tumor with acanthosis, hyperkeratosis and parakeratosis; (2) identical WC between penile, vulva and uterine cervix or anus; (3) arborescent papillary pattern with long, rounded or spiky papillae with prominent fibrovascular cores; (4) conspicuous koilocytosis, increased nuclear size with hyperchromasia, wrinkling and bi- or multinucleation, perinuclear halos and individual cell necrosis, throughout entire tumor; (5) possibility of intraepithelial abscesses; (6) sharply delineated interface between tumor and stroma with no invasion in early stage (noninvasive WC); and (7) jagged boundary between tumor and stroma in later stage (invasive WC) [4, 9].

The treatment of WC could depend on tumor size, body site, surgical tools or equipment, etc. Nowadays, systemic chemotherapy is not broadly used for treating SCCs including WC. As a result, the fundamental principle is complete removal of the tumor.

In conclusion, although WC usually occurs on genito-urethral and perineal area, this case shows that it can arise from the subcutaneous tissue on the back. Pathological differentiation is significant because there are a variety of SCCs. Any tumor of the elderly should be thoroughly evaluated in addition to physical examination, past medical and social history, underlying diseases, and other preoperative blood tests since early detection and treatment is important for these patients. Consequently, we should keep delicate wide excision, maintaining the safety margin in surgery and periodic follow-up in outpatient clinic.
